# Brivaracetam in the Treatment of Patients with Epilepsy—First Clinical Experiences

**DOI:** 10.3389/fneur.2018.00038

**Published:** 2018-02-06

**Authors:** Felix Zahnert, Kristina Krause, Ilka Immisch, Lena Habermehl, Iris Gorny, Izabella Chmielewska, Leona Möller, Anna M. Weyand, Peter M. Mross, Jan Wagner, Katja Menzler, Susanne Knake

**Affiliations:** ^1^Department of Neurology, Universitätsklinikum Gießen und Marburg, Philipps University of Marburg, Marburg, Germany

**Keywords:** brivaracetam, levetiracetam, epilepsy, treatment, side effects

## Abstract

**Objectives:**

To assess first clinical experiences with brivaracetam (BRV) in the treatment of epilepsies.

**Methods:**

Data on patients treated with BRV from February to December 2016 and with at least one clinical follow-up were collected from electronic patient records. Data on safety and efficacy were evaluated retrospectively.

**Results:**

In total, 93 patients were analyzed; 12 (12.9%) received BRV in monotherapy. The mean duration to follow-up was 4.85 months (MD = 4 months; SD = 3.63). Fifty-seven patients had more than one seizure per month at baseline and had a follow-up of more than 4 weeks; the rate of ≥50% responders was 35.1% (*n* = 20) in this group, of which five (8.8%) patients were newly seizure-free. In 50.5% (47/93), patients were switched from levetiracetam (LEV) to BRV, of which 43 (46.2%) were switched immediately. Adverse events (AE) occurred in 39.8%, with 22.6% experiencing behavioral and 25.8% experiencing non-behavioral AE. LEV-related AE (LEV-AE) were significantly reduced by switching to BRV. The discontinuation of BRV was reported in 26/93 patients (28%); 10 of those were switched back to LEV with an observed reduction of AE in 70%. For clinical reasons, 12 patients received BRV in monotherapy, 75% were seizure–free, and previous LEV-AE improved in 6/9 patients. BRV-related AE occurred in 5/12 cases, and five patients discontinued BRV.

**Conclusion:**

BRV seems to be a safe, easy, and effective option in the treatment of patients with epilepsy, especially in the treatment of patients who have psychiatric comorbidities and might not be good candidates for LEV treatment. BRV broadens the therapeutic spectrum and facilitates personalized treatment.

## Introduction

More than 30% of patients with epilepsy are refractory to medication with antiepileptic drugs (AEDs) (1, 2), although pharmaceutical treatment options have expanded steadily over the last 20 years. While many AEDs have similar potency with regard to seizure control, they often vary with regard to tolerability and side effects, which have the most important impact on treatment compliance and thus on seizure control (3, 4). Brivaracetam (BRV) is the latest AED, which was approved in Germany in February 2016 as an adjunctive treatment of partial-onset seizures with and without secondary generalization in patients aged 16 years or older (5). Similar to levetiracetam (LEV), it mainly targets synaptic vesicle protein 2A, but more selectively and with a 15- to 30-fold increased affinity (6, 7). Clinical trials have demonstrated a significant reduction of seizure frequency after the initiation of BRV, with ≥50% responder rates ranging from 30.3 to 55.8% (8–15). At a dose of 100 mg/day, the amount of seizure-free patients was sevenfold compared to placebo (16). During those trials, the overall efficacy of BRV was greater in LEV-naïve patients. In previous clinical studies, treatment-emergent adverse events (AE) of BRV occurred in 54.2% of the patients. The most common AE were irritability, fatigue, asthenia, somnolence, headache, paresthesia, and dizziness (13, 16–18). Compared to LEV, the occurrence of AE and seizure control was similar, with a significantly higher incidence of dizziness in BRV (18). However, behavioral adverse events (BAE) are common in LEV (19) and they accounted in one study for 40.4% of discontinuations of LEV therapy (3). In the therapeutic range of 50–200 mg BRV per day, BAE such as anxiety, aggression, depression, or others occurred in 5.0–12.3% (5). In a small, open-label prospective exploratory study, a direct switch from LEV to BRV led to a reduction of BAE in 27/29 patients, making BRV a promising treatment option in patients with LEV-associated BAE (20).

So far, long-term post-marketing observations can provide further important insight into the efficacy and tolerability under real-life conditions. Here, we report post-marketing experience with BRV.

## Patients and Methods

Data were retrospectively collected from in- and outpatients of the Epilepsy Center Hessen, Germany, who received BRV treatment after its approval and introduction to the German market between February 2016 and December 2016. All patients who had at least one clinical follow-up were included. Based on a decision of the local IRB, patients do not have to be consented for retrospective data analysis.

The effect of BRV on seizure frequency and tolerability was assessed. Data were collected at baseline (BL) (i.e., initiation on BRV) and at each of the follow-up visits, usually after 3 and 6 months. Data on concurrent anticonvulsant medication, seizure frequency, initiation, and termination of treatment as well as AE were identified from electronic patient records.

Responder rates were assessed at the most recent follow-up in patients on BRV who had seizure frequencies of ≥1 per month at BL and a follow-up of at least 4 weeks. Patients were classified as seizure-free when no seizures occurred during the entire observation period.

Analyses of AE were separated into two different subgroups: behavioral AE, including psychiatric AE such as depression and anxiety, and non-behavioral AE, including AE such as dizziness, cognitive decline, and others.

To compare the tolerability of LEV and BRV, we analyzed the reoccurrence and persistence of LEV-associated AE, which either had emerged on LEV in medical history, leading to the discontinuation of LEV, or were present on LEV at BL. The number of LEV-related AE (LEV-AE) was compared with the number of the same LEV-AE reoccurring under BRV in each respective patient using repeated measures *t*-tests.

## Results

A total of 93 patients who received BRV during the observation period and who had at least one follow-up visit were identified and included in the analysis.

### Demographic Characteristics

For demographic characteristics of the study population, see Table 1. In the subpopulation eligible for the analysis of seizure frequencies, the mean length of the observation period was 5.3 months (MD = 5.5 months, SD = 4.1 months). A direct switch from LEV monotherapy to BRV monotherapy was performed in 12 patients (12.9%). Patients had received 6.3 AEDs on average since their first diagnosis of epilepsy (MD = 5 AEDs, SD = 3.7). Four (4.3%) patients were initiated on a further AED other than BRV within the observation period.

**Table 1:** Patients’ characteristics.

Characteristic	Baseline (BL),*n* = 93
Age (years), M (SD)	43.9 (17.3)
Sex, *n* (%)	
– Male	58 (62.4)
– Female	35 (37.6)
Epilepsy duration (years), M (SD)	19.3 (14.7)
Epileptic seizure profile	
– Idiopathic generalized, *n* (%)	3 (3.2)
– POS, *n* (%)	90 (96.8)
Period of follow-up (months), M (SD)	4.85 (3.6)
Number of previous AEDs, M (SD)	6.3 (3.7)
Number of AEDs concomitant to BRV, M (SD)	1.7 (1)
Psychiatric comorbidity	42 (45.2)

**BRV daily dose in mg**	***n* = 93 (%)**

50	26 (28)
100	55 (59.1)
150	4 (4.3)
200	8 (8.6)

**Concomitant AEDs in >5% of patients**	***n* = 93**

Lamotrigine	32
Lacosamide	25
Valproate	17
Zonisamide	14
Oxcarbazepine	13
Topiramate	12
Perampanel	8
Pregabalin	5
Vagus nerve stimulation	7
BRV monotherapy	12

**LEV medication at BL**	***n* = 93 (%)**

LEV intake at BL	47 (50.5)
– immediate switch to BRV	43 (46.2)
– gradual switch	4 (4.3)
LEV in (past) medical history	87 (93.5)
LEV naïve	6 (6.5)

### Seizure Frequencies and Responder Rates

**Figure 1:**
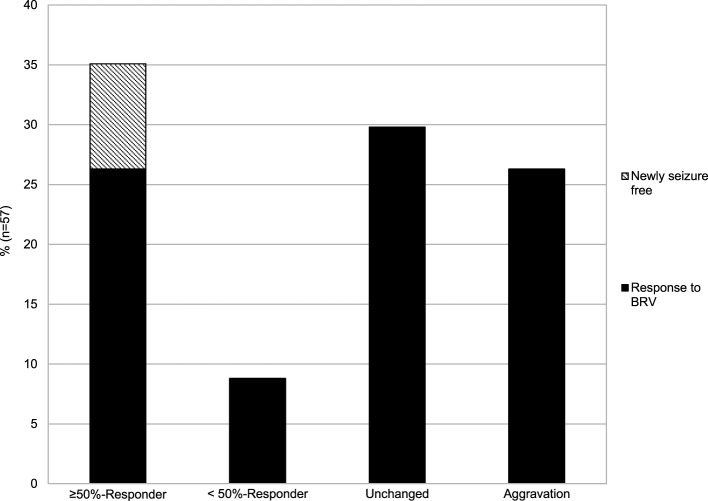
Change in seizure frequency after initiation of BRV treatment.

Responder rates at the most recent follow-up (average 5.3 months after first BRV prescription) were determined in 57/93 patients (Figure 1). The rate of ≥50% responders was 35.1% (*n* = 20), of which five (8.8%) patients became seizure-free. The rate of <50% responders was 8.8% (*n* = 5), while seizure frequency remained unchanged in 29.8% (*n* = 17). An aggravation of seizure frequency occurred in 26.3% (*n* = 15) of patients. Overall seizure freedom was achieved in 27/93 patients (29%). Status epilepticus was observed in four (4.3%) patients under BRV treatment.

Due to sample size, we did not investigate seizure frequencies and responder rates in LEV-naïve patients (*n* = 6) separately.

### Treatment-Emergent AE and Discontinuation Rates

Overall, AE during BRV intake occurred in 37 (39.8%) patients. BAE and NBAE were observed in 21 (22.6%) and 24 (25.8%) patients, respectively.

Adverse events reported in >5% of patients were irritability, depression, fatigue (*n* = 7, 7.5%), aggression (*n* = 6, 6.5%), and cognitive decline (*n* = 5, 5.4%). For other AE, see Table 2.

**Table 2:** The number of total AE during BRV therapy (overall AE under BRV) and the number of AE that were associated with previous LEV treatment at BL (LEV-AE at BL) and under treatment with BRV (^a^LEV-AE on BRV at the most recent follow-up).

AE	Overall AE on BRV,*n* = 93 (%)	LEV-AE at BL, *n* = 36 (%)	LEV-AE on BRV^a^,*n* = 36 (%)
Drug-related AE	37 (39.8)	36 (100)	12 (33.3)
Behavioral AE	21 (22.6)	31 (86.1)	10 (27.8)
Irritability	7 (7.5)	9 (25)	3 (8.3)
Depression	7 (7.5)	10 (27.8)	3 (8.3)
Aggression	6 (6.5)	9 (25)	3 (8.3)
Agitation	2 (2.2)	5 (13.9)	2 (5.6)
Psychosis	2 (2.2)	3 (8.3)	1 (2.8)
Listlessness	1 (1.1)	2 (5.6)	0 (0)
Anxiety	1 (1.1)	1 (2.8)	0 (0)
Lability of affect	1 (1.1)	0 (0)	0 (0)
Hysteria	1 (1.1)	0 (0)	0 (0)

Non-behavioral AE	24 (25.8)	12 (33.3)	3 (8.3)
Fatigue	7 (7.5)	6 (16.7)	1 (2.8)
Cognitive deficit	5 (5.4)	4 (11.1)	2 (5.6)
Dizziness	3 (3.2)	2 (5.6)	1 (2.8)
Sleep disturbance	3 (3.2)	3 (8.3)	0 (0)
Reduced consciousness	1 (1.1)	0 (0)	0 (0)
Weight loss	1 (1.1)	0 (0)	0 (0)
Other	12 (12.9)	2 (5.8)	1 (2.8)

*Multiple indication were possible*.

*Note that some patients suffered multiple AE. Cognitive deficit was not objectified and mirrors subjective impressions by the patient. AE classified as “other” were non-severe NBAE, respectively, occurring in one patient only*.

The discontinuation of BRV was reported in 26/93 patients (28%). Reasons for the discontinuation of BRV are listed in Table 3. Two patients (2.2%) requested to discontinue AED therapy entirely. The mean duration from BRV initiation to discontinuation was 3.19 months (MD = 3 months, SD = 2.6). The most frequent AE leading to discontinuation was aggression (*n* = 4, 4.3%).

**Table 3:** Reason for discontinuation of BRV therapy.

Discontinuation due to	*n* = 93 (%)
AE	19 (20)
– BAE	12 (12.9)
– NBAE	11 (11.8)
Lack of seizure control	14 (15.1)
– Status epilepticus	4 (4.3)
Wish to discontinue therapy at all	2 (2.2)

In 12/15 patients (80%) who had a follow-up after BRV discontinuation, a clinically meaningful reduction of AE was observed.

An immediate switchback to LEV was performed in 10/26 patients who discontinued BRV. Of these patients, eight were followed up: seven (87.5%) of those showed clinical improvement.

### LEV-Associated AEs

More than half of the patients (57/93) had AEs under LEV treatment before, either in their prior medical history (*n* = 21), or at actual BL (*n* = 36). In this population, 44/57 (77.2%) patients reported either a clinically meaningful reduction or no reemergence of previous LEV-AE under BRV at all.

**Figure 2:**
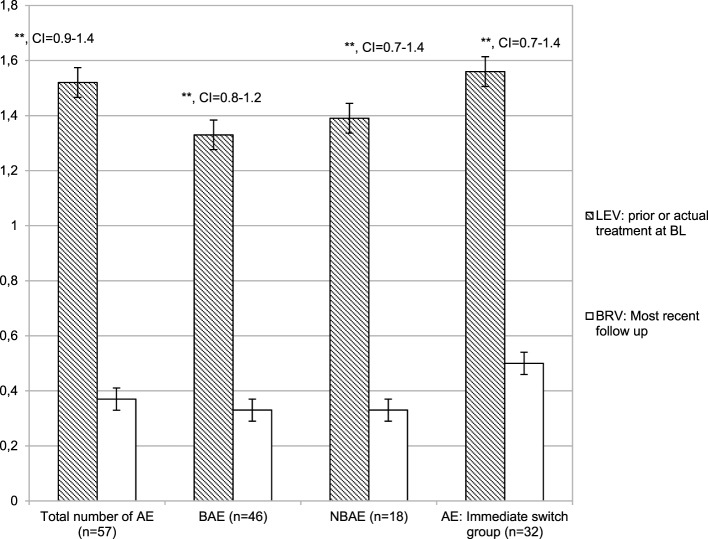
Number of adverse events: [total number and number of behavioral and non-behavioral adverse events (AE)] under Levetiracetam (LEV) treatment and under brivaracetam (BRV) treatment in the subpopulation of patients who ever (actual or in prior treatment) had AE under LEV medication. Thirty-two patients had been switched immediately from LEV to BRV. **p < 0.001, and error bars indicate the mean error.

Out of 36 patients suffering from LEV-AE at BL, 24 (66.67%) experienced a clinically meaningful reduction in AE by switching to BRV. Comparison of the mean number of LEV-AE at BL and the most recent follow-up on BRV for each respective patient revealed a significant reduction of an average of 1.08 AE (*p* < 0.001, M_1_ = 1.56, SD_1_ = 0.91; M_2_ = 0.47, SD_2_ = 0.81) (Figure 2).

The mean number of LEV-AE was reduced significantly in patients who were immediately switched from BL on LEV to BRV (*n* = 32) (*p* < 0.001, M_1_ = 1.56, SD_1_ = 0.95; M_2_ = 0.5, SD_2_ = 0.84).

Significant reductions in LEV-BAE were observed between BL on LEV and the most recent follow-up on BRV (*n* = 31; *p* < 0.001, M_1_ = 1.26, SD_1_ = 0.63; M_2_ = 0.39, SD_2_ = 0.67), and a similar reduction was observed in patients experiencing LEV-NBAE at BL (*n* = 12; *p* = 0.001, M_1_ = 1.4, SD_1_ = 0.67; M_2_ = 0.42, SD_2_ = 0.9). The aggravation of LEV-AE occurred in four patients (4.3%).

Patients who were not currently on LEV treatment, but who had discontinued LEV in the past due to AE (*n* = 21), reported a significantly smaller amount of those LEV-associated AEs after being treated with BRV (*n* = 21; *p* < 0.001, M_1_ = 1.4, SD_1_ = 0.75; M_2_ = 0.19, SD_2_ = 0.51). Here, similar results emerged regarding BAE (*n* = 15; *p* < 0.001, M_1_ = 1.47, SD_1_ = 0.74; M_2_ = 0.2, SD_2_ = 0.56) and NBAE (*n* = 6; *p* = 0.013, M_1_ = 1.33, SD_1_ = 0.52; M_2_ = 0.17, SD_2_ = 0.41). Only 2/21 (9.5%) patients experienced a reoccurrence of LEV-associated AE.

### Monotherapy

Within our study population, 12/93 patients received BRV in monotherapy, based on individual therapeutic decisions and on individual medical reasons. Here, the mean observation period was 4.6 months (SE = 0.8, SD = 2.7; median = 4 months) and the mean number of AEDs prescribed in prior anamnesis was 2.1 (SE = 0.3, SD = 1.2, median = 2).

In this subpopulation, freedom from seizures was achieved in 9/12 (75%) patients.

In 6/9 (66.67%) patients, LEV-AE from BL were reduced to a clinically and statistically significant extent (n = 9; *p* = 0.011, M_1_ = 2.3, SD_1_ = 1; M_2_ = 1, SD_2_ = 1.3). Overall, AE occurred in 5/12 patients (41.7%), the most common (in >10%) being irritability and agitation (*n* = 2, 16.7%).

Five of these 12 patients (41.7%) discontinued therapy with BRV, with a mean duration of therapy until discontinuation of 3.65 months (SE = 1.5, SD = 3.3, median = 3.5 months). Two patients (16.7%) discontinued due to BAE, and two stated that they wished to discontinue therapy entirely. Non-behavioral AE accounted for three discontinuations. Again, multiple, simultaneously occurring AE leading to discontinuation were common.

## Discussion

Our primary objective was to assess seizure control and tolerability in patients under BRV treatment.

We found that treatment with BRV can effectively reduce seizure frequency in patients with epilepsies with a ≥50% responder rate of 35.1 and 8.8% of patients being newly seizure-free.

Data are consistent with results from earlier trials (27.8–55.8%), and our rate of patients newly free of seizures (8.8%) was within the range of previously described rates (3–14.9%) (8–14, 21, 22). An exacerbation of seizure frequency occurred in 26.3%. This rate may not be surprising, considering the highly selective group of patients with a long history of treatment-resistant epilepsy resulting in a comparatively high number of previously prescribed AEDs. Responder rates on BRV were comparable to post-marketing experiences with other recently introduced AEDs such as Perampanel (PER; ≥50% responder rate in the largest three trials: 27–50%) or Lacosamide (LCM; ≥50% responder rate: 18–69%). The same did also apply to rates of seizure-free patients (PER: 14–17% in the largest three trials; LCM: 3–33%), as in the present study, 29% were seizure-free with 8.8% being newly seizure-free (23, 24).

Within our study population, AE on BRV were common and occurred in 39.8% of patients, which is consistent with the findings of a retrospective clinical study (37%) and slightly lower than indicated by the findings of a pooled analysis of phase IIb and phase III trials (54.2%) (13, 21).

In a meta-analysis of previous clinical trials, AE significantly associated with BRV compared to placebo were somnolence, dizziness, fatigue, and irritability, with an incidence of 12.4, 9.6, 7.7, and 2.8%, respectively (16). In another pooled analysis, the most common AE overall were headache (20.9%), dizziness (17.5%), somnolence (15.2%), nasopharyngitis (13.2%), fatigue (11.3%), and convulsion (10.6%) (13). In this study, the safety profile of BRV differed from the above data. While in previous studies, the most common BAE (irritability, insomnia, depression, and anxiety) occurred in only 2–3% of patients (16), BAE emerged more frequently under BRV (22.6%) in our study population. In our analysis, depression, irritability and fatigue (7.5% each), and aggression (6.5%) were the most frequently reported AEs. Dizziness, one of the most common AEs from previous studies, only occurred in 3.2%, and somnolence was observed in only one patient (1.1%). One major reason for the higher rate of BAE in our data might be that BRV was initiated in patients who mostly had psychiatric comorbidities (45.2%) or who were prone to behavioral side effects and had already discontinued LEV due to BAE. Unlike some other AEDs, BRV did not cause metabolic syndrome or weight gain (25).

As previous studies suggested, the effects on seizure frequency seemed strongest in patients who were LEV-naïve (16). Hence, the safety and efficacy of BRV administered in monotherapy is of great interest. The administration of BRV as the first anticonvulsive treatment in patients is yet to be examined. In our data, monotherapy with BRV appeared safe and was well tolerated with a reduction of LEV-associated AE in the majority of patients, supporting previously described experiences (22). Patients on monotherapy had less severe epilepsy and were previously on another monotherapy. Switching to BRV was mainly performed due to behavioral side effects or psychiatric comorbidities and not due to a lack of seizure control. This explains the greater proportion of seizure-free patients than in the overall study population.

In patients who were switched from LEV to BRV, a reduction of AE was observed. AEs, which had led to LEV discontinuation in the past, rarely reemerged under therapy with BRV.

Our findings indicate that BRV has a safety profile that is distinct from LEV, making it a useful alternative to enhance adherence to therapy with AEDs. Especially for patients who are not eligible for LEV use, BRV might be a therapeutic option, opening a chance to achieve sufficient seizure control.

These results are consistent with the findings from previous studies where a reduction of LEV-associated BAE was described (20, 22).

An immediate switch from LEV to BRV was safe and reduced LEV-associated AE in the majority of patients. Switching back from BRV to a prior anticonvulsive medication, especially LEV, was safe, and AEs as well as increases in seizure frequency emerging under BRV seem at least partially reversible this way. Due to the sample size, further studies investigating the pharmacokinetics and the clinical impact of a fast and direct switch of anticonvulsive medication are of interest.

### Limitations

The use of retrospective data obtained by a review of the patient charts and from a standard patient anamnesis in daily clinical practice might potentially introduce individual bias. This might stem from the neurologists’ individual evaluations and interpretations, as well as the variable comprehensiveness of patient self-reports. However, these results mirror conditions in clinical practice, where the clinician mostly relies on patient self-report, and standardized data are not always available.

Results in analyses for subgroups such as patients on monotherapy or immediate switchback to LEV might consolidate with reanalysis once more data become available. Larger, prospective, and multicenter trials of these subgroups would be desirable.

## Conclusion

Therapy with BRV seemed safe and well tolerated. An immediate switch from LEV to BRV was easy and safe and reduced LEV-associated AE. However, behavioral and non-behavioral AEs occurred under BRV treatment. In case of newly occurred AE on BRV, a direct switchback to LEV was safe. Single patients were treated for individual reasons with BRV monotherapy, which seemed safe and achieved seizure freedom in 9/12 patients.

In summary, we demonstrated that BRV might be a promising option for the treatment of epilepsies, especially for those patients who suffer from side effects of LEV therapy. BRV seems to offer the chance to improve therapeutic effectiveness and broadens the therapeutic spectrum to facilitate personalized treatment.

## Ethics Statement

For the local IRB, the ethics committee of the Department of Medicine, Philipps University Marburg, extra ethical approval is not needed, if data are collected retrospectively; any patients in our University Hospital agree upon admission that data might later be used anonymously for research purposes.

## Author Contributions

FZ contributed to conception, acquisition of data, analysis and interpretation of data, and manuscript drafting. KK participated in data analysis, manuscript drafting, and revision. II, IC, JW, LH, AS, IG, AW, PM, and LM contributed to data collection and manuscript revision. KM contributed to study conception, data analysis, and draft writing. SK contributed to study conception, data analysis, and draft writing.

## Conflict of Interest Statement

FZ, KK, LH, IG, IC, LM, AW, and JW—none; II served as a study investigator for UCB, SAGE Therapeutics, Boehringer Ingelheim, Marinus Pharmaceuticals Inc., Upsher-Smith Laboratories Inc., GlaxoSmithKline, Eisai Limited. She received speaker’s honoraria for UCB. KM worked as a Consultant for UCB and Eisai. SK served on advisory boards for UCB and held lectures for UCB, Desitin and Eisai. She is study investigator for Sage, UCB and Boehringer Ingelheim, Marinus Pharmaceuticals Inc., Upsher-Smith Laboratories Inc., GlaxoSmithKline and Eisai Limited.
